# Natural killer cells drive 4-1BBL positive uveal melanoma towards EMT and metastatic disease

**DOI:** 10.1186/s13046-023-02917-5

**Published:** 2024-01-09

**Authors:** Shi Yong Neo, Mariana M. S. Oliveira, Le Tong, Yi Chen, Ziqing Chen, Sonia Cismas, Nutsa Burduli, Anna Malmerfelt, Joey Kay Hui Teo, Kong-Peng Lam, Evren Alici, Leonard Girnita, Arnika K. Wagner, Lisa S. Westerberg, Andreas Lundqvist

**Affiliations:** 1 Singapore Immunology Network (SIgN), Agency for Science, Technology and Research (A*STAR), Singapore, Republic of Singapore; 2 Department of Oncology-Pathology, Karolinska Institutet, Stockholm, Sweden; 3 Department of Microbiology, Tumor and Cell Biology, Karolinska Institutet, Stockholm, Sweden; 4 Department of Medicine, Division of Hematology and Oncology, Columbia University Irving Medical Center, New York, USA; 5 Department of Molecular Biology, Lewis Thomas Laboratory, Princeton University, Princeton, NJ USA; 6 Department of Medicine Huddinge, Karolinska Institutet, Stockholm, Sweden

**Keywords:** Natural killer cells, Uveal melanoma, Epithelial-to-mesenchymal transition

## Abstract

**Background:**

Inflammation in the eye is often associated with aggravated ocular diseases such as uveal melanoma (UM). Poor prognosis of UM is generally associated with high potential of metastatic liver dissemination. A strong driver of metastatic dissemination is the activation of the epithelial-mesenchymal transition (EMT) regulating transcription factor ZEB1, and high expression of ZEB1 is associated with aggressiveness of UM. While ZEB1 expression can be also associated with immune tolerance, the underlying drivers of ZEB1 activation remain unclear.

**Methods:**

Transcriptomic, in vitro, ex vivo, and in vivo analyses were used to investigate the impact on clinical prognosis of immune infiltration in the ocular tumor microenvironment. A metastatic liver dissemination model of was developed to address the role of natural killer (NK) cells in driving the migration of UM.

**Results:**

In a pan-cancer TCGA analysis, natural killer (NK) cells were associated with worse overall survival in uveal melanoma and more abundant in high-risk monosomy 3 tumors. Furthermore, uveal melanoma expressed high levels of the tumor necrosis factor superfamily member 4-1BB ligand, particularly in tumors with monosomy 3 and BAP1 mutations. Tumors expressing 4-1BB ligand induced CD73 expression on NK cells accompanied with the ability to promote tumor dissemination. Through ligation of 4-1BB, NK cells induced the expression of the ZEB1 transcription factor, leading to the formation of liver metastasis of uveal melanoma.

**Conclusions:**

Taken together, the present study demonstrates a role of NK cells in the aggravation of uveal melanoma towards metastatic disease.

**Supplementary Information:**

The online version contains supplementary material available at 10.1186/s13046-023-02917-5.

## Background

Uveal melanoma (UM) is a unique and rare form of melanoma occurring in the uveal tract of the eye. In general, the poor prognosis of UM is associated with the high potential of metastatic dissemination to the liver. Unlike cutaneous melanoma where BRAF mutations are common, therapeutic responses to targeted therapies against UM remain poor. The majority of UM cases are associated with activating mutations of GNAQ/GNA11 that trigger oncogenic signaling [1, 2]. However, several reports showed no significant associations between these mutations with clinical prognosis [3, 4]. One possible explanation for limited response of metastatic UM to therapeutic strategies targeting Gαq/11 is high redundancy of hyperactivated signaling pathways. Hence, the current standard care for UM are mainly localized radiotherapy, resection or enucleation surgery, and close follow up for metastatic disease development [5]. In this context, biomarkers to understand UM pathogeny and progression, and tools to predict metastatic potential are essential. Over the last three decades, prognostic factors for metastatic UM include increasing age, elevated levels of LDH, alkaline phosphatase, size of liver lesions, as well as pathological characteristics of the primary tumor including size, location, shape, and patterns formed by malignant cells [5].

An important development of UM prognostication was the recognition of monosomy 3 as the most prominent cytogenetic marker associated with liver metastasis. Moreover, it is now well-established that inactivating mutation of BRCA1 associated protein-1 (BAP-1), which is located on chromosome 3p21.1, is a critical event resulting in enhanced metastatic potential of UM cells [6]. Subsequently, several molecular strategies were developed including the pivotal study by Onken *et. al.* classifying UM into two molecular subclasses based on gene expression profiling (GEP) of a set of twelve genes [7, 8]. Presently used to aid for the diagnosis and monitoring of UM, independent or associated with the loss of chromosome 3 and BAP-1 mutation, UMs can be classified as class 1, (2–21% 5-year metastatic risk) or class 2, (72% 5-year metastatic risk) [9].

Another strong driver of metastasis is the activation of ZEB1, which is one of the transcription factors regulating epithelial-mesenchymal transition (EMT) of UM. Moreover, ZEB1 has been associated with therapeutic resistance in cancer which can be counteracted by HDAC inhibitors such as mocetinostat [10]. High expression of ZEB1 is associated with tumor aggressiveness of UM [11]. While several studies reported that ZEB1 tumor expression is also associated with immune tolerance including the accumulation of suppressive macrophages and CD8 T cell exhaustion [12, 13], the underlying drivers of ZEB1 activation remain unclear. Despite low immune infiltrates within UM, our pan-cancer analysis revealed high expression of 4-1BBL in tumors associated with molecular risk factors of metastatic disease such as monosomy 3 and GEP class 2 phenotype.

Although natural killer (NK) cells are best known for their antineoplastic capacity, the potential of NK cells to display immune regulatory properties has recently been highlighted. We previously identified NK cells to upregulate the ectonucleotidase CD73 upon ligation of 4-1BB during tumor-NK cell interaction. These NK cells showed increased capacity to inhibit T cell responses [14]. Furthermore, recent observations have demonstrated that NK cells can influence metastatic dissemination [15, 16]. Here we extend these findings and demonstrate that ligation of 4-1BB induces NK cells to promote ZEB1 activation and increase liver metastasis of UM. Further analysis revealed that such pro-metastatic NK cells upregulated CD73 surface expression, accompanied with metabolic and inflammatory transcriptomic re-programming.

## Methods

### Public transcriptomic datasets

The TCGA pan-cancer data, including ACC, BLCA, BRCA, CCA, CESC, CHOL, COAD, DLBC, ESCA, GBM, HNSC, KICH, KIRC, KIRP, LAML, LGG, LIHC, LUAD, LUSC, MESO, OV, PAAD, PCPG, PRAD, READ, SARC, SKCM, TGCT, THCA, THYM, UCEC, UCS, and UVM were downloaded from Xena browser (https://xenabrowser.net/datapages/). The scaled estimate values of fragments per kilobase of exon model per million reads mapped (FPKM) were converted to transcripts per million (TPM) values by multiplying by one million. Three different NK gene signatures were obtained from previous studies [17–19]. Kaplan–Meier analysis, log-rank test, and Cox proportional hazards regression model were conducted by R package "survminer" and "survival". Tumor-infiltrating lymphocyte (TIL) signature score was defined by ssGSEA method. Gene enrichment was performed based on several gene set databases such as KEGG, msigDB hallmarks, Reactome and gene ontology (GO). GSEA software (mSigDB) was used for gene set enrichment analysis on mSigDB hallmark gene sets.

For the access and subsequent analysis of publicly available single cell sequencing dataset for UM tumors (GSE139829), BBrowser software (Bioturing) was used for uMAP clustering analysis and then to compare TNFSF9 expression based on the original author’s pre-defined cell subtypes together with other phenotypic parameters such as GEP and PRAME expression status. For NK cell and tumor sub-clustering analysis, we used Partek Flow software (Partek) to first identify NK cells (*CD3E*-, *CD3D*-, *CD14*-, *NKG7* + and *PTPRC* + cells) and tumor cells (*MITF* + *, MLANA* +). Normalized gene counts were subsequently analyzed using Louvain clustering, monocle 2 for trajectory analysis and differential gene expression with hierarchical clustering using the hurdle model.

### Tumor cell lines

OCM-1, OCM-3 and MEL270 were kindly provided by Martine Jager, Leiden University Medical Center, Leiden, The Netherlands, previously described [20–22]. All cell lines were propagated and maintained in RPMI supplemented with 10% FBS. Validation of cell lines by short tandem repeat profiling of extracted genomic along with testing for Mycoplasma contamination DNA was performed (Eurofins Genomics) once per year, prior to cryopreservation of stocks. To establish stable mCherry-expressed cell lines, the third-generation lentiviral transduction system was used.

The pLV-mCherry was a gift from Pantelis Tsoulfas (Addgene plasmid # 36,084; http://n2t.net/addgene:36084; RRID: Addgene_36084). pLV-mCherry was co-transfected with plasmids expressing virus coat and assembly proteins (REV, RRE, and VSVG) into 70 to 80% confluent HEK293T cells using Lipofectamine 3000 reagent (Life Technologies). Conditioned media were collected after 24, 48 and 72 h, filtered through 0.45 μm low protein binding membranes (Sarstedt) and later concentrated at 3200 g for 15 min. The supernatant medium containing virus particles was stored at -80 degree for future use.

The generation of 4-1BBL CRISPR knockout cell lines were previously described in a previous study [14]. In brief, 1.5X10^4^ of either OCM-1 or OCM-3 cells suspended in 2 mL of RPMI medium supplemented with 10% FBS were transduced with 500 μL of virus-containing supernatant for 8 h in 8 μg/mL protamine sulfate (Sigma-Aldrich). After transduction, cells were cultured for at least 14 days prior to FACS-sorting based on 4-1BBL surface expression on FACSAria™ fusion (BD) Sorted cells were sub-cultured and propagated for at least two passages before use for subsequent experiments.

### NK cell isolation

Human peripheral blood samples were obtained from anonymized donors from the Karolinska University Hospital Blood Bank. Peripheral blood mononuclear cells were collected through Ficoll density gradient centrifugation (GE Healthcare). Primary NK cells were isolated by negative selection following the manufacture protocol (Miltenyi Biotec, Human NK cells isolation kit). Isolated NK cells were cultured in X-vivo 20 medium (Lonza) supplemented with 10% heat-inactivated human AB serum and 100 IU/mL of IL-2 (Proleukin) for 48 h before subsequent co-culture experiments.

### Generation of liver organoids

Anonymized liver tissues were obtained from Karolinska Hospital Huddinge. Liver single cell suspensions were generated by following human Tumor Dissociation Kit (Miltenyi Biotec) protocol. The suspension was filtered through a 40 μm Nylon cell strainer and centrifuged for 4 min at 500g. Liver organoids were cultured as previously published [23]. Briefly, the cell pellet was washed with cold Advanced DMEM/F12 and spun at 500g for 4min. Single cells were mixed with 50% Advanced DMEM/F12 (GIBCO) and 50% Matrigel (Corning) on ice at the density of 40,000 cells per 100 μl solution, and 50 μl mixture was seeded per well to pre-warmed 24 well plate (TPP) to form the dome. The plate was then placed in 37℃ incubator for 15 min. Organoid culture medium was added after Matrigel had solidified. Organoids culture medium components are shown in Supplemental Table 1. As it was previously published, Nicotinamide was identified as an inhibitory factor that reduced NK cell cytotoxicity during colon organoids and NK-92 co-culture, and nicotinamide-free medium didn’t influence normal colon organoids growth in three days [24]. Therefore, Nicotinamide was excluded from the medium when culturing liver organoids with NK cells. Organoids were passaged every 10–12 days. For freezing organoids, organoids were mechanically dissociated into small fragments and mixed with freezing medium CryoStor® CS10 (StemCell Technologies) and frozen. Organoids were thawed using standard procedures, before being cultured as described above, organoids were washed with Advanced DMEM/F12 supplemented with 1% BSA, GlutaMAX, HEPES, and Penicillin–Streptomycin.

### Microscopy

For 3D immunofluorescence and confocal imaging, liver organoids were fixed with 4% Paraformaldehyde (Sigma) for 1 h at 4°C. Organoids were then blocked in DPBS with 0.2% Triton X-100 (Sigma), 10% DMSO (Sigma), 6% Bovine Serum Albumin (BSA, Sigma) overnight. Antibodies were diluted in DPBS with 5% DMSO and 3% BSA. Details of antibodies and dilution are shown in Supplemental Table 2. Samples were then incubated with primary antibodies at 37℃ for 1 day. After being washed with DPBS three times, samples were next incubated with secondary antibodies and YO-PRO-1 (ThermoFisher Scientific) at 37℃ for 1 day. Afterwards, organoids were washed in DPBS for three times. All steps including blocking, antibody incubation, and washing were done on a shaker at 37℃. Samples were observed under ZEISS LSM 800.

### Immunohistochemistry

The collection and use of human samples in accordance with the Declaration of Helsinki was approved by the ethics committee of the Karolinska Institutet (2016/658–31/2). For hematoxylin staining and immunohistochemistry (IHC), PFA-fixed tissues were transferred to 70% ethanol overnight and then filtered through a funnel with a 5 × 5cm silk filter paper before formalin fixation. The formalin fixed samples were processed in an automated tissue processing machine (LOGOS, Milestone) and embedded in paraffin. 4-μm-thick FFPE sections (formalin-fixed, paraffin-embedded) were mounted on Superfrost + glass slides (Thermo Scientific) and heated for 3 h at 56°C. After de-paraffinization in xylene and rehydration in alcohol, a HIER was performed using a Decloaking Chamber (Biocare Medical) set for 5 min at 110^O^C in Citrate buffer pH 6 (Sigma, C-9999). For quenching of endogenous peroxidase, a 30-min incubation in 0,15% hydrogen peroxidase was performed at room temperature, followed by a 30 min blocking step using 1% Bovine Serum Albumin (Sigma A-4503). The primary antibody 4-1BBL (#PA5-47,291, ThermoFisher) were diluted 1/200 in Renior Red diluent (Biocare Medical #PD9004M), incubation at 4 °C overnight in a humid chamber. For detection, a rabbit anti goat AP conjugated antibody was used (Invitrogen, A16139) diluted 1/500 in TBS with 0.2% Tween for 30 min, visualized by DAKO Liquid Permanent Red K0640 with incubation time of 10 min. The sections were counterstained in Mayer´s hematoxylin for 1 min followed by brief dehydration: three rapid dips in 99.5% ethanol, three rapid dips in xylene and cover slipped with Pertex (Histolab). For hematoxylin and eosin staining, slides also undergo de-paraffinization in xylene and rehydration in alcohol. Slides were then stained with Mayer´s HTX (5 min), rinsed in water (5 min), followed by distilled water. Next, 0.2% Eosin (Histolab) was used to stain the slides for 75 s, followed by dehydration with graded alcohols, xylene and cover slipped with Pertex (Histolab).

### Transwell migration assay

In vitro migration assays were performed with GFP-expressing OCM-3 cells with or without 41BBL-KO. 3 × 10^5^ tumor cells were seeded in a transwell inserts of 8μm pore size (Corning) in serum-free RPMI media before the addition of 1 × 10^5^ NK cells in suspension. The transwell inserts containing both NK and tumor cells were then transferred into a 24 well plate containing 10% FBS in RPMI media cultured in 37°C, 5% CO_2_ incubator for 48 h. Subsequently, the transwell inserts with media removed, rinsed with PBS and then fixed with 4% paraformaldehyde (PFA) solution. Using a cotton swab, cells on the inner side of the transwell inserts were physically removed prior to imaging of the outer transwell membrane under 5 × magnification on ZIESS LSM 800 confocal system. Quantification of GFP positive tumor cells on the outer membrane was done on IMARIS software (Bitplane, Oxford Instruments).

### Quantitative reverse transcription polymerase chain reaction

Cells were lysed using TRIzol (Life Technologies) and phenol–chloroform extraction was performed to isolate total RNA. Complementary strand of DNA was generated using RevertAid First Strand cDNA Synthesis Kit (Thermo Fisher Scientific). The following primers were used to run real-time PCR using SYBR Green PCR Master Mix (Applied Biosystems): ZEB1 (forward): CGAGTCAGATGCAGAAAATGA; ZEB1 (reverse): ACCCAGACTGCGTCACATGTC; SIAH1 (forward): TCTTCCTGGTGCTGTTGACTGG; SIAH1 (reverse): CGATTGCGAAGAACTGCTGGTG; GAPDH (forward): TCAAGGCTGAGAACGGGAAG; GAPDH (reverse): CGCCCCACTTGATTTTGGAG.

### Real-time cytotoxicity assay

In a 96-well plate, target parental OCM-3 cells and 41BBL-KO OCM-3 cells were co-cultured with effector NK cells at an effector to target ratio of 5:1 in RPMI with the caspase-3/7 viability dye diluted 1:1000 (Thermo Scientific). The 96-well plate was then incubated in Incucyte S3 realtime imaging system over a duration of 48 h set with fluorescent images acquired every 4 h. The data was analyzed on the Incucyte software using the cell-by-cell module.

### Flow cytometry

Single cell suspensions were first washed with FACS wash buffer (5%FBS in PBS) before incubation with surface antibodies for 20 min in 4°C. For staining of intracellular markers ZEB1 and Ki67, the eBioscience™ FOXP3/ Transciption Factor Staining buffer set was used according to manufacturer’s recommended protocol. All intracellular antibody mix were incubated with the cells for 30 min in room temperature. List of all FACS antibodies used were provided as Supplemental Table 3. After cells were washed with FACS wash buffer, all samples were then acquired on Novocyte Quanteon (Agilent) and subsequently analyzed on Flowjo software (BD).

### In vivo experiments

In vivo xenograft studies were conducted in accordance with the approved animal ethical permit (11159-2018). In-house bred NOD-scid-gamma (NSG) mice used in this study were 8 weeks old of age and all male gender. 1 × 10^6^ IL-2 activated NK cells were infused via the tail vein into the NSG mice prior to the subcutaneous injection of 5 × 10^6^ of either parental or 41BBL-KO mCherry OCM-1 cells into the right flank of the mice. Tumor volume was monitored and measured over an experimental duration of 24–30 days. After experimental endpoint, all mice were sacrificed for harvesting of tumor and liver tissues for subsequent FACS analysis and IHC. For IHC, fixed and paraffin embedded mouse liver tissue sections were processed for immunostaining antigen retrieval for 20 min at 100 °C using EDTA buffer, pH 9. Slides were incubated with blocking serum (1% BSA) for 20 min followed by incubation with primary antibodies against the established markers used for human melanoma detection, NuMa (Abcam, ab84680) or HMB45 (Agilent, M063429-2) overnight at 4°C and later with secondary antibody for 1h at room temperature. Tissue sections were also counterstained with hematoxylin and rinsed with deionized water. The Bond III automated stainer for immunohistochemistry/Chromogenic in situ hybridization (IHC/CISH, Leica, Wetzlar, Germany) was used for performing deparaffinization, pre-treatment, primary and secondary staining, and counterstaining. The SLIDEVIEW VS200 research slide scanner was used to scan the slides and the OlyVia 3.1 software (Olympus Corporation, Tokyo, Japan) for analysis.

### Zebrafish xenografts

MEL270 cells that were pre-labelled with red fluorescent DIL (Thermo Fisher) and human NK cells were mixed at a 1:3 ratio (MEL270: NK). Zebrafish embryos at the age of 24 hpf (hours post fertilization) were incubated in water containing 0.2 mmol/L 1-phenyl-2-thio-urea (PTU, Sigma). At 48-hpf prior to microinjection, zebrafish embryos were dechorionated and anesthetized with 0.04 mg/mL of tricaine (MS-222, Sigma). The microinjection of human cell mixture was performed by infusing 5nL (approximately 500 cells in total) into the perivitelline space of each larvaes using an Eppendorf microinjector (FemtoJet 5247, Eppendorf and Manipulator MM33-Right, Märzhäuser Wetziar). Successfully injected larvae were transferred into PTU aquarium water at 33°C for 48 h incubation before fixation with 4% paraformaldehyde (PFA) for image acquisition. 3D Images of zebrafish larvaes were acquired on Thunder Imaging System (Leica Microsystems) under 4X objectives. Batch quantification of different treatment groups were done using IMARIS software. (Bitplane, Oxford Instruments).

### Statistics

All experimental data were plotted and tested for significance using Prism 8.0 (GraphPad Software) as described in figure legends unless stated otherwise. *P* values below 0.05 were considered significant. All error bars represent SD of the mean.

## Results

### NK cell gene signature correlates with worse prognosis in uveal melanoma

**Fig. 1:**
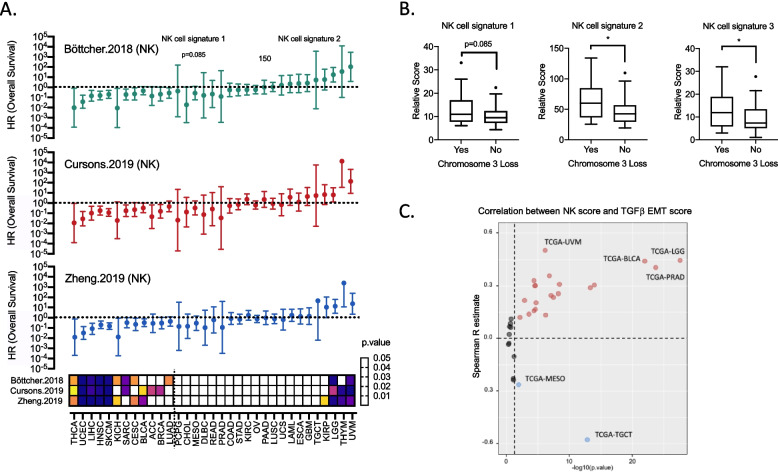
NK cell gene signature correlates with worse prognosis in uveal melanoma **A** Forest plots for three NK cell gene signatures and their influence on overall survival in 33 different cancer types across TCGA-Pan cancer database. Heatmap shows respective *p*-values based on cox proportional hazards model. **B** Relative scores for the three different NK cell gene signatures comparing UM samples with or without the loss of chromosome 3. Mann–Whitney was used to test for significance (*n* = 80). Signature 1: Böttcher 2018, signature 2: Cursons 2019, and signature 3: Zheng 2019. **C** Volcano plot showing correlation and significance between NK cell gene signature (Böttcher 2018) with a TGFβ-EMT gene signature in the 33 different cancer types of TCGA-Pan cancer cohort. Spearman correlation was used for every cancer cohort of the TCGA dataset. Cancer types with significant spearman *R* value of > 0.4 or < -0.4 were highlighted

Several recent studies have devised various versions of NK cell gene signatures to explore how their expression influences prognosis of patients with different cancer types. Comparing three published NK cell gene signatures showed in general similar prognostic outcomes across the pan-cancer TCGA cohort [17–19], whereby high expression was associated with significantly prolonged overall survival in 13 solid tumor types including cutaneous melanoma (SKCM), liver cancer (LIHC) and head and neck squamous carcinoma (HNSC). In contrast, high NK cell signature scored for worse overall survival in low-grade glioma (LGG) and uveal melanoma (UVM) (Fig. 1A). All three NK signatures were associated with poor survival in patients with UM with hazard ratios of 3.183, 4.811 and 5.939 respectively (Supplementary Fig. 1). Notably, these NK signatures correlated with the monosomy 3 phenotype, which is a classical molecular feature driving tumor progression (Fig. 1B).

Applying a previously reported gene signature for EMT [17] in the TCGA pan-cancer cohort revealed a strong correlation of NK cell and TGF-EMT gene signature scores in UM (Fig. 1C). Additionally, the TGF-EMT signature was found to be prognostic for worse overall and progression free survival (Supplementary Fig. 2). Three additional tumor types, bladder cancer (TCGA-BLCA), prostate adenocarcinoma (TCGA-PRAD), and low-grade glioma (TCGA-LGG) also showed a high positive correlation of NK cell and EMT gene signature scores with a spearman R of more than 0.4. Of note, NK cell signature negatively correlated with EMT signatures in mesothelioma (TCGA-MESO) and testicular germ cell tumors (TCGA-TGCT) (Fig. 1C). While there is a greater appreciation for how NK cell abundance is important for anti-tumor immunity, our pan-cancer analysis provided indications that high NK cell abundance may contribute to metastatic dissemination of UM.

### 4-1BBL is highly expressed in uveal melanoma and associated with worse prognosis

**Fig. 2:**
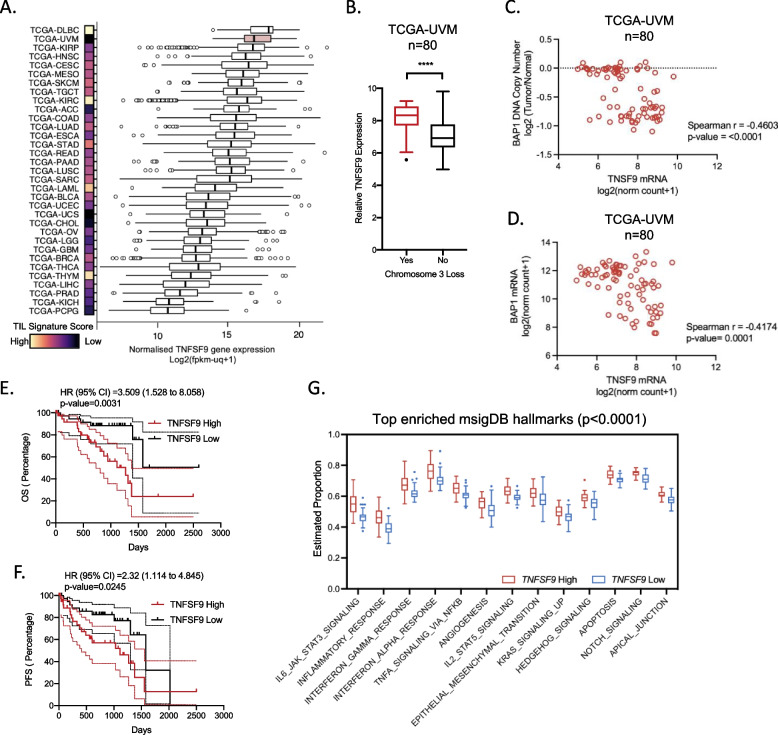
4-1BBL is highly expressed in uveal melanoma and is prognostic for worse survival **A** Tukey Boxplots showing normalized gene expression of TNFSF9 across 33 cancer types of the TCGA-Pan cancer dataset together with a heatmap comparing the relative TIL signature score defined by ssGSEA. The UM cohort (TCGA-UVM) is highlighted as a red boxplot. **B** Relative TNFSF9 gene expression within the TCGA-UVM cohort comparing tumors with or without loss of chromosome 3. Mann–Whitney was used to test for significance (*n* = 80). **C** and **D** Correlation between TNFSF9 gene expression with (**C**) BAP1 DNA copy number and (**D**) BAP1 gene expression. Correlations were analyzed with Spearman’s test. **E** and **F** Kaplan–Meier plots of the TCGA-UVM cohort (*n* = 80) grouped based on the median gene expression of TNFSF9 for (**E**) overall survival (OS) and (**F**) Progression-Free survival (PFS). Log-rank test was used to test for hazard ratios and significance. **G** Tukey Boxplots showing significantly enriched (*p* < 0.0001) mSigDB hallmarks gene signatures comparing 2 groups of TCGA-UVM tumor cases (*n* = 80) grouped based on the median gene expression of TNFSF9

We previously identified that NK cells upregulate the expression of CD73 and acquire immune regulatory properties via the interaction with 4-1BBL on tumor cells [14]. Although UM has a low tumor-infiltrating lymphocytes (TIL) signature score, it is ranked second highest across all solid tumors in the TCGA pan-cancer cohort in terms of 4-1BBL expression (Fig. 2A). Similar to the expression of NK gene signatures, the expression of *TNFSF9* is significantly higher in tumors with loss of chromosome 3 (Fig. 2B). Furthermore, significant negative correlations are observed between *TNFSF9* expression and *BAP1* DNA copy number and *BAP1* gene expression (Fig. 2C and D). Notably, *TNFSF9* is prognostic for worse overall and progression-free survival (Fig. 2E and F). Using mSigDB hallmarks which is a curated database of refined gene sets [25], we furthermore, found that high *TNFSF9* expression is associated with higher enrichment score for several tumor-promoting hallmarks gene sets including inflammation, angiogenesis and EMT (Fig. 2G). Collectively, pan-cancer analysis identifies UM as a unique cancer type in which 4-1BBL expression is strongly associated with unfavorable tumor molecular features and clinical prognosis.

### Presence of NK cells promote migration of uveal melanoma cells into liver organoids

**Fig. 3:**
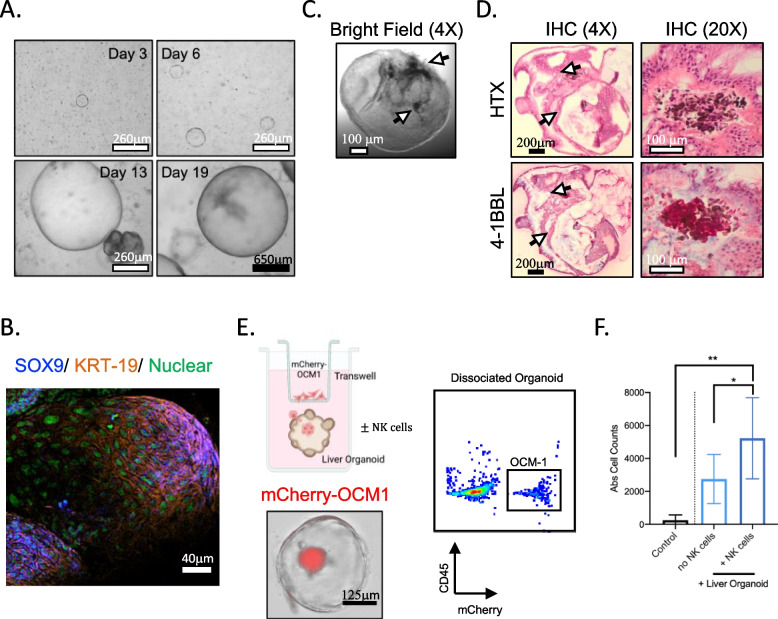
NK cells promote migration of uveal melanoma cells into liver organoids **A** Representative bright field images of liver tissue-derived organoids visualized at day 3, 6, 13 and 19 under 4X objective. Black and white scale bars denote 650μm and 260μm respectively. **B** Representative confocal image showing the expression of SOX9 and KRT-19 within liver organoid under 10X objective. White scale bar denotes 40μm. **C** Bright field image of a liver organoid with pigmented primary UM cells under 4X objective. White scale bar denotes 100μm. **D** Immunohistochemistry of liver organoid containing a cluster of pigmented UM tumor cells shown from hematoxylin (HTX) staining and 4-1BBL expression via IHC at 4X and 20X objective. Black and white scale bars denote 200μm and 100μm respectively. **E** Experimental setup of transwell-organoid metastasis in vitro model, representative brightfield image and flow cytometric plot of liver organoid containing mCherry + OCM-1 tumor cells. Scale bar denotes 125μm. **F** Absolute cell counts of mCherry tumor cells based on flow cytometry of dissociated organoids with or without presence of NK cells. One-way ANOVA with multiple comparison was used to test for significance (*n* = 5)

Based on observations from TCGA transcriptomics analysis, we sought to address if NK cells impact metastatic potential of UM. Using a well-established protocol for generating liver organoids [23], we first validated that cultured cells generated 3D structures (Fig. 3A), and the expression of SOX9 and Keratin-19 (KRT-19) (Fig. 3B). Addition of primary UM tumor cells (pigmented melanocytes) (Fig. 3C) and strong expression of 4-1BBL resulted in the formation of micrometastases within the organoid microenvironment (Fig. 3D). Using this model, the presence of NK cells resulted in significantly higher frequency of UM cells within the liver organoids as analyzed by brightfield imaging and flow cytometry of mCherry-expressing OCM-1 UM cells (Fig. 3E and F). To our knowledge, these results are the first to demonstrate that NK cells promote tumor metastasis into liver organoids.

### Activation of ZEB1 and tumor migration is dependent on the expression of 4-1BBL and presence of NK cells

**Fig. 4:**
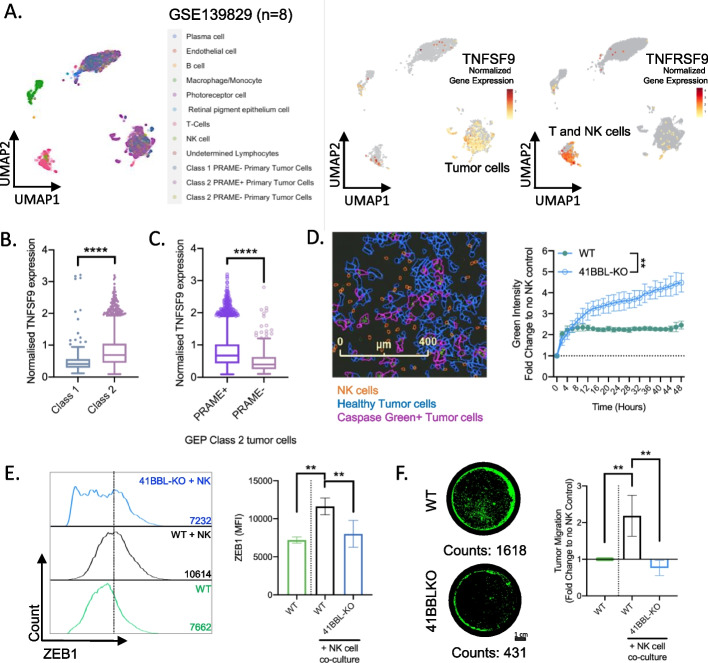
Activation of ZEB1 and tumor migration is dependent on 4-1BBL expression and NK cells **A** uMAP projection of single cell transcriptomics from primary UM tumors (GSE139829, *n* = 8) showing the expression profile of TNFSF9 and TNFRSF9 in different cell types. **B** Normalized gene expression of TNFSF9 comparing tumor cells of GEP class 1 over class 2. Mann–Whitney was used to test for significance. **C** Normalized gene expression of TNFSF9 comparing GEP class 2 tumor cells that are PRAME + over PRAME- cells. Mann–Whitney was used to test for significance. **D** LEFT: Representative image of realtime NK cell cytotoxicity captured at 48 h. RIGHT: Realtime NK cell cytotoxicity assay over 48 h showing relative increase in green fluorescence due to dye detecting activated caspase-3/7 in tumor cells. Two-way ANOVA was used to test for significance between the two conditions. **E** LEFT: Representative flow histogram of ZEB1 expression. RIGHT: Mean fluorescence intensity (MFI) of ZEB1 protein expression in parental wild type (WT) and 41BBL-KO OCM-3 cells after 3 days of NK cells co-culture. **F** LEFT: Representative image of transwell migration showing GFP-OCM3 cells under 5X objective magnification. Scale bar denotes 1 cm. RIGHT: Relative migration of WT and 41BBL-KO OCM-3 tumor cells. Prior to the 48 h transwell migration assay, tumor cells were co-cultured for 3 days with NK cells before harvested for assay. **E**, **F**, One-way ANOVA was used to test for significance (*n* = 4)

Single-cell transcriptomic analysis was recently used to study tumor heterogeneity and to identify putative immune checkpoint targets of UM [26]. Using this publicly available dataset (GSE139829), we investigated the potential role of 4-1BBL in development of metastatic disease. As visualized by uMAP analysis, *TNFSF9* is mainly expressed in primary tumors whereas *TNFRSF9* is expressed within T and NK cell clusters (Fig. 4A). To further address the prognostic value of *TNFSF9*, UM tumors were stratified based on unfavorable molecular characteristics such as GEP class 2 and PRAME expression. This analysis revealed that GEP class 2 tumors show significantly higher levels of *TNFSF9* (Fig. 4B). While PRAME is a driver for genomic instability and recognized as a strong predictor for metastasis for metastatic disease [27, 28], the expression of *TNFSF9* is significantly higher in PRAME positive GEP class 2 tumors (Fig. 4C).

To investigate if NK cells impact on the metastatic potential of tumor cells and if ligation of 4-1BB is involved in this process, a 4-1BBL knockout (KO) OCM-3 tumor cell line was established. While wild-type (WT) OCM-3 cells are considerably resistant to NK cell-mediated cytotoxicity, 4-1BBL KO OCM-3 cells are significantly more susceptible to killing by NK cells (Fig. 4D). Furthermore, NK cells induced the expression of ZEB1 in WT but not 4-1BBL KO OCM-3 cells (Fig. 4E). The biological impact of ZEB1 expression was tested in transwell migration assays, demonstrating that NK cells induced migration of WT OCM-3 but not 4-1BBL KO OCM-3 cells (Fig. 4F). While previous studies demonstrated that ZEB1 is also regulated by ubiquitination [29–31], we explored if NK cell-driven ZEB1 upregulation could be due to protein stabilization and prevention of ubiquitination-directed degradation. Using the publicly available single cell dataset (GSE139829), we performed an unsupervised clustering of tumor cell from primary tumors to first identified 16 subclusters for differential gene expression analysis. Notably, clusters 12 and 14 are cells derived from 2 separate patients with known metastasis (Supplementary Fig. 3A). Unsupervised heatmap clustering revealed cluster 12 and 14 to have close homology in terms of the high expression of USP51 and CASP8AP2 which are both known to promote ZEB1 protein stabilization [30, 31] (Supplementary Fig. 3B). Comparing non-metastatic tumor cluster 1 versus either cluster 12 or 14, we not only found lower expression of TNFSF9 (encoding 41BBL) but also higher levels of SIAH1 which is an E3 ligase that was found to be downregulated during TGFB-induced EMT [29] (Supplementary Fig. 3B). To validate our findings, we further demonstrated that tumor cells not only transcriptionally upregulated ZEB1 but also downregulated SIAH1 upon exposure to NK cells (Supplementary Fig. 3C and D). Collectively, these results demonstrate that 4-1BBL is critical to the pro-metastatic interaction between tumor and NK cells.

### NK cells promote liver metastasis of 4-1BBL-expressing uveal melanoma

**Fig. 5:**
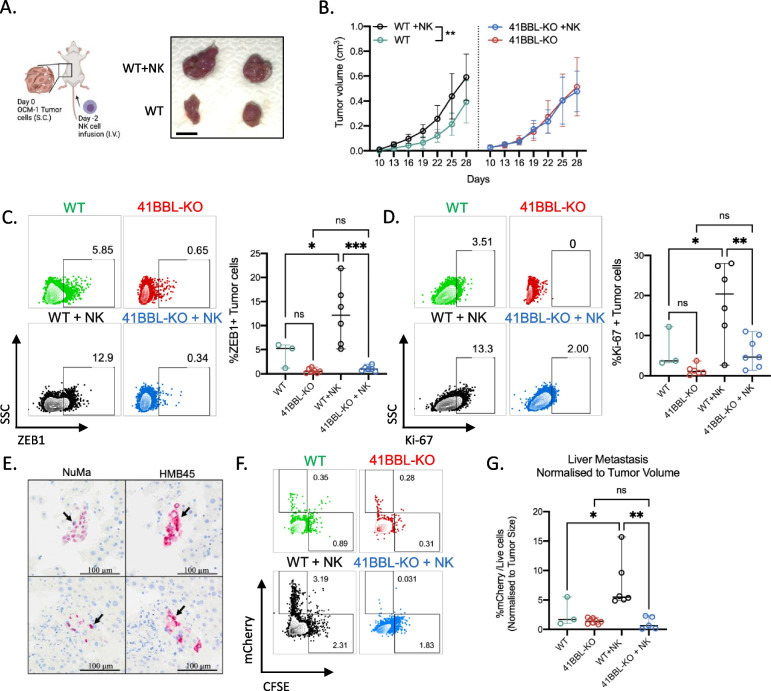
NK cell promotes liver metastasis of 4-1BBL-expressing uveal melanoma **A** Schematic illustration of xenograft experimental setup using NSG mice and image of representative mCherry + OCM-1 xenograft tumors harvested from either NSG infused with NK cells or control naïve NSG mice. **B** Growth of WT and 41BBL-KO OCM-1 tumors in NK cell-infused or naïve mice over a period of 28 days. Two-way ANOVA was used to test for significance (*n* = 7). **C** and **D** Representative histograms and Flow cytometry analysis for frequencies of ZEB1 + (**C**) and Ki67 + (**D**) OCM-1 cells harvested from WT and 41BBL-KO tumors in naïve and NK cell-infused mice. One-way ANOVA with multiple comparisons was used to test for significance. **E** Immunohistochemistry of mouse liver sections containing UM micro-metastases expressing human NuMA and HMB45. **F** Representative flow cytometry plots of mCherry + OCM-1 cells detected in cell suspensions of livers from tumor bearing mice with or without NK cell infusion. **G** Frequencies of mCherry + tumor cells in the liver normalized to primary tumor volume in mice with or without NK cell infusion. One-way ANOVA with multiple comparisons was used to test for significance

Next, we sought to investigate if NK cells influence progression of primary tumors and metastatic liver dissemination of UM in vivo (Fig. 5A, left). While tumor size of primary WT OCM-1 xenografts is significantly larger in mice treated with NK cells, no difference in tumor size of primary 4-1BBL-KO OCM-1 tumors is observed between untreated and NK cell-treated mice (Fig. 5A, right and 5B). Notably, upon infusion of NK cells, WT xenografts show higher frequencies of ZEB1 and Ki67 positive tumor cells compared with 4-1BBL-KO xenografts (Fig. 5C and D). Importantly, we further validated that NuMA (a marker for human cells) and HMB45 positive micrometastases are detected in livers of tumor-bearing mice (Fig. 5E). Flow cytometry quantification of mCherry + tumor cells show significantly higher metastatic dissemination of WT but not 4-1BBL-KO tumor cells in mice infused with NK cells (Fig. 5F and G). Collectively, these findings demonstrate that 4-1BBL expression on tumor cells and NK cells contribute to the development of liver metastasis of UM cells.

### NK cells undergo phenotypic changes to acquire inflammatory features within the uveal tumor microenvironment

**Fig. 6:**
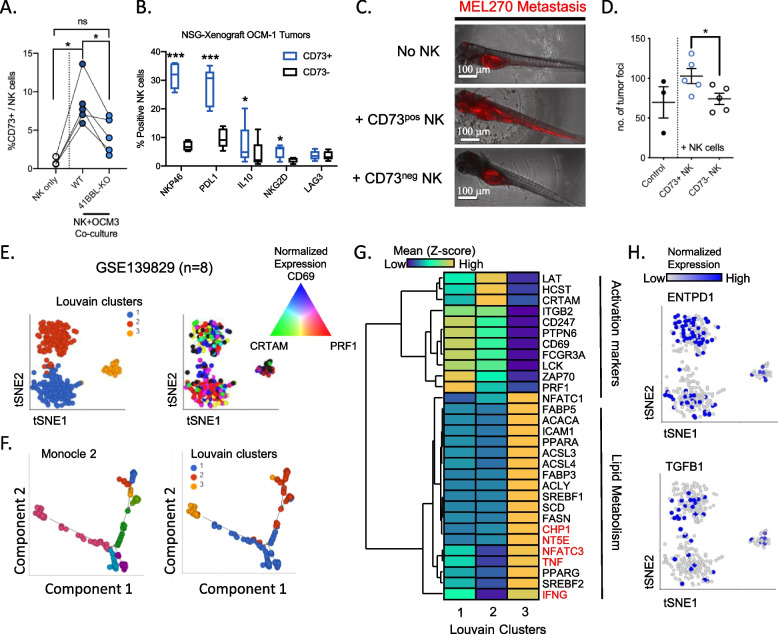
Characterization of CD73 positive pro-metastatic NK cells in uveal melanoma **A** Frequencies of CD73 positive NK cells after 4 h co-culture with either parental OCM-3 or 4-1BBL-KO OCM-3. Wilcoxon signed-rank test was used to test for significance (*n* = 5). **B** Proportions of CD73 positive versus negative NK cells expressing NKp46, PD-L1, IL-10, NKG2D and LAG-3. Mann–Whitney test was used to determine the significance (*n* = 4). **C** Representative images of red fluorescent labelled MEL270 localization within zebrafish larvae. White scale bar denotes 100μm. **D** Number of tumor foci quantified within every zebrafish larva. Student t-test was used to determine significance comparing xenografts injected with either CD73 positive or negative NK cells (*n* = 5). E) tSNE projection of NK cells from primary uveal melanoma tumors (GSE 139829, *n* = 8) colored based on Louvain clusters (left) and the normalized gene expression of *CRTAM, CD69* and *PRF1*. **F** Trajectory analysis using monocle 2 algorithm which reveals transitional subclusters (left) in relation to Louvain clusters (Right). **G** Hierarchical heatmap clustering of differentially expressed genes comparing the 3 louvain clusters. Genes in red font are upregulated in cluster 3 but not related to lipid metabolism. H) tSNE projection of NK cells and their normalized expression of *ENTPD1* (top) and *TGFB1* (Bottom)

Aligned with our previous findings showing that CD73 surface expression is induced upon ligation of 4-1BB and characterizes immune regulatory functions in NK cells [14], we found that the expression of CD73 is induced by parental OCM-3 cells and not 4-1BBL KO OCM-3 cells (Fig. 6A). Furthermore, CD73 positive NK cells co-express NKp46, PD-L1, IL-10 and NKG2D as compared with CD73 negative NK cells (Fig. 6B). Given the immune regulatory role of CD73 positive NK cells, the ability of these cells to enhance metastatic potential of tumor cells was investigated. Due to a low cell recovery of CD73 positive NK cells, the zebrafish larvae were used as a model of tumor dissemination. Co-injection of CD73 positive NK cells and tumor cells promoted the formation of metastases from the perivitelline space to the tail (Fig. 6C and D).

To identify NK cell phenotypic alterations associated with their pro-metastatic role in UM, single cell transcriptomic dataset of primary uveal melanoma tumors (GSE139829) was analyzed. Using Louvain clustering, three distinct clusters of NK cells were identified of which only clusters 1 and 2 expressed higher levels of *PRF1, CRTAM and CD69* (Fig. 6E). Trajectory analysis further revealed additional transitional cell states in relation to the three Louvain clusters (Fig. 6F). In addition, a significant downregulation of several immune effectors and activation markers was observed in cell cluster 3 compared with the other two clusters. Notably in cluster 3, we observed a striking upregulation of genes related to lipid metabolism together with the increased expression of *NT5E* (encoding CD73), *TNF, IFNG, CHP1 and NFATC3* (Fig. 6G). On the other hand, NK cells from these uveal melanoma tumors were found to express *CD39* and *TGFB1* which are not exclusively expressed within the CD73 positive cluster 3 (Fig. 6H). Taken together our data demonstrate tumor-induced reprogramming of CD73 positive NK cells leading to their pro-tumoral function.

## Discussion

Although the eye is considered an immune-privileged site, the current understanding of the immune microenvironment of the eye is the presence of myeloid cells, NK and even resident memory T cells [32]. Within its unique immune landscape, high concentrations of TGFβ and immune-suppressive neuropeptides can convert conventional T cells into Tregs [33]. Similar to trophoblast and neuronal cells, multiple cell types in the eye within the cornea, iris and retina express low levels of MHC molecules [32]. When exposed to pro-inflammatory cytokines such as IL1β, IFNγ and TNFα, retinal pigment epithelium (RPE) decline in its physiological functions due to transcriptomic alterations which in turns upregulate EMT-promoting genes such as ZEB1 and SNAI1 [34]. In autoimmune ocular conditions such as squamous metaplasia, IL1β and IFNγ trigger CREB and ZEB1 activation that upregulate SPRR1B expression that is involved in the pathological keratinization of the epithelium [35]. It is conceivable that the flux of inflammatory immune cells influences UM progression and metastatic dissemination. The abundance of immune cells per se would result in better prognosis in cancer as exemplified in many studies using TCGA datasets. However, our study identifies UM as a unique cancer in which immune infiltration implicates unfavorable clinical outcome. A recent study that conducted whole genome sequencing of tumors and characterization of TILs, revealed unique molecular driver events during tumor progression, and marked immune evasion despite the presence of tumor-reactive TILs [36]. Thus, tumor immune escape in the eye could potentially be attributed by non-canonical immune suppression mechanisms.

Recent studies demonstrate that the immunoregulatory roles of NK cells is attributed to its cytotoxic functions against other immune cells and production of inflammatory cytokines to modulate the tumor microenvironment [37, 38]. Even though NK cells are best recognized for their cytolytic capacity, their ability to secrete cytokines such as IFNγ and TNFα may act as potential drivers of tumor progression. Moreover, the capacity of NK cells to produce inflammatory cytokines is associated with impaired cytotoxicity [39, 40]. The potential of NK cells to secrete these inflammatory factors could be governed by tumor MHC class I expression [41, 42]. Our previous study defined a particular immune-suppressive subset of NK cells that express CD73 and at the same time, produce IL-10 and TGFβ [14]. Given that IL-10 and TGFβ modulate tumor cell biology such as EMT, it is plausible that these CD73 positive NK cells within the tumor microenvironment influence the metastatic potential of tumor cells. Recent studies have provided insights into how NK cells could acquire a pro-metastatic cell state during immune tolerance and tumor progression [15, 16].

Our previous study identified the induction of CD73 on NK cells upon 4-1BB ligation during tumor-NK cell interaction. The present study reveals that UM has the highest median expression of 4-1BBL out of 33 cancer types despite being a cancer within an immune privileged site. Further analysis into public single cell transcriptomic dataset validated that UM tumor cells express 4-1BBL and its gene expression is associated with the unfavorable molecular characteristics such as GEP class 2, PRAME expression, loss of chromosome 3 and BAP1 mutation.

Here we show that, 4-1BBL expression is strongly associated with molecular signatures favoring metastasis and is prognostic for worse survival in UM. Yet, the role of 4-1BB stimulation within the eye remains elusive. While 4-1BB is a cancer immunotherapy target used in CAR-T cell and monoclonal antibodies modalities, an early study showed that agonistic 4-1BB antibody could trigger immune-suppression and alleviate an autoimmune disease in the eye known as experimental autoimmune uveoretinitis (EAV) [43]. Likewise, we observed that 4-1BB activation may not stimulate anti-tumoral immune responses, but rather immune-regulatory and pro-metastatic functions mediated by NK cells. A previous study mechanistically demonstrated the phosphorylation at Thr-867 site triggers ZEB1 activity implicating EMT [44]. Even though we demonstrate that the presence of NK cells activated ZEB1 in UM cells both in vitro and in vivo, the underlying mechanism in which these pro-metastatic NK cells triggered EMT and tumor invasiveness remain unclear. Choi et al. demonstrated that the 4-1BB ligation stimulates IFNγ production to suppress EAV [43]. Notably, 4-1BB-mediated immune suppression is absent in IFNγ-KO mice suggesting an unconventional role of 4-1BB/IFNγ axis in repressing immune responses. Although the findings by Choi are relevant in the context of 4-1BB-mediated immune suppression, we did not explore the relative contribution of IFNγ by NK cells in dissemination of UM. Considering the recent clinical development of agonizing 4-1BB in cancer immunotherapy [45], it is interesting to understand if such approach impacts on the ability of NK cells to affect EMT. Our present study identified NK cell mediated upregulation of ZEB1 in UM cells which implicate positive regulation of EMT. ZEB1 contributes to immune evasion via reduction of CD8 T cells infiltration in cutaneous melanoma and lung cancer[12]. Moreover, ZEB1-mediated tumor progression was only prominent in immunocompetent murine models [46]. Another study reported that ZEB1-expressing tumor cells drive the development of a suppressive immune microenvironment at the invasive front via the upregulation of arginase and PD-L1 during M2 macrophage polarization [13]. Given that IFNγ activates ZEB1 [35, 47], future studies are motivated to address if the metastatic potential of UM is triggered by such soluble inflammatory factors or if a single critical factor could independently trigger an unique intrinsic signaling pathway downstream of 4-1BBL expressed on tumor cells.

A previous study in 1997 reported that the aqueous humor within the anterior chamber of the eye constitutes an immune suppressive milieu that inhibits NK cell-mediated cytotoxicity against UM and even MHC class I-deficient RMA-S cells implanted within the eye microenvironment [48]. Based on our transcriptomics analysis, we further investigated the biology of NK cells within UM tumors, identifying metabolic shifts to be associated with the immune-regulatory phenotype and impaired anti-tumor cytotoxicity functions. To date, the influence of metabolic changes on intratumoral NK cell functions remain poorly understood. Our earlier study reported IL-15 to promote glycolysis in NK cells in a mTOR dependent manner together with enhanced effector functions [49]. A more recent study showed that activation via NKG2D or CD16 engagement upregulates glycolysis and OXPHOS and that the inhibition of glycolysis dampens degranulation and killing capacity of NK cells [50]. Our present study reports that NK cells with impaired cytotoxicity displayed altered lipid metabolism. However, what stimulatory cues from the tumor microenvironment trigger such metabolic changes in intratumoral NK cells remain elusive. Future studies to comprehensively investigate metabolic activity of tumor-infiltrating NK cells and more importantly, targeting such critical pathways to reinvigorate these NK cells for antitumor functions are warranted. Taken together, while the presence of NK cells restricts primary tumor growth, NK cells may contribute to inflammatory disease and drive tumor progression in terms of metastatic potential.

## Conclusions

Although NK cells are recognized for their anti-tumor activity, recent studies underline their potential in driving tumor progression. Here we demonstrate that NK cell frequency and high expression of 4-1BB ligand is associated with poor prognosis in UM. Upon ligation of 4-1BB, NK cells induce the activation of the epithelial-mesenchymal transition regulating transcription factor ZEB1 in UM, resulting in increased formation of liver metastasis. Further analysis revealed an upregulation of CD73 surface expression, accompanied with metabolic and inflammatory transcriptomic re-programming in NK cells. Our results identify a previously unknown property of NK cells to acquire a pro-metastatic function and highlights the need to comprehensively understand the plasticity of NK cells and beyond their role in immune surveillance of cancer. With such knowledge, improved therapies based on the activation of NK cells can be developed for solid cancers.

### Supplementary Information

**Additional file 1: Supplementary Figure 1.** Prognostic values of NK cell signatures on overall survival of uveal melanoma patients. **Supplementary Figure 2.** Prognostic values of a TGF-EMT signature on survival of uveal melanoma patients. **Supplementary Figure 3.** Gene expression profiling of uveal melanoma cells. **Supplemental Table 1.** Components for liver organoid culture media. **Supplemental Table 2.** Antibodies for Immunofluorescence. **Supplemental Table 3.** Antibodies for Flow Cytometry.

## Data Availability

The datasets used and/or analyzed during the current study are available from the corresponding author on reasonable request.

## Abbreviations

NK: Natural Killer
UM: Uveal Melanoma
BAP-1: BRCA1 associated protein-1
GEP: Gene expression profiling
EMT: Epithelial-mesenchymal transition
GO: Gene ontology,
TIL: Tumor-infiltrating lymphocyte
KO: Knockout
WT: Wild-type
