# Supplementary material for: Natural killer cells drive 4-1BBL positive uveal melanoma towards EMT and metastatic disease
Source: J Exp Clin Cancer Res. 2024 Jan 9;43:13. doi: 10.1186/s13046-023-02917-5 (PMC10775428; doi:10.1186/s13046-023-02917-5)
Supplement: Supplementary file 1 — Additional file 1: Supplementary Figure 1. Prognostic values of NK cell signatures on overall survival of uveal melanoma patients. Supplementary Figure 2. Prognostic values of a TGF-EMT signature on survival of uveal melanoma patients. Supplementary Figure 3. Gene expression profiling of uveal melanoma cells. Supplemental Table 1. Components for liver organoid culture media. Supplemental Table 2. Antibodies for Immunofluorescence. Supplemental Table 3. Antibodies for Flow Cytometry. [file 13046_2023_2917_MOESM1_ESM.docx]

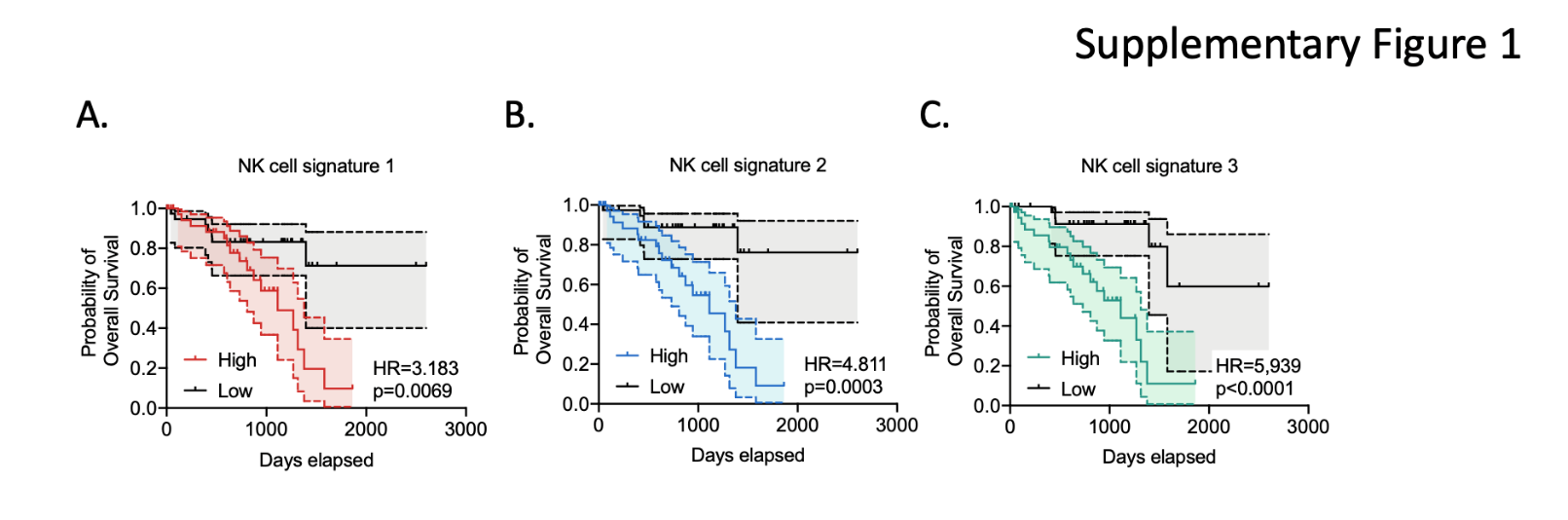
Supplementary figure 1. Prognostic values of NK cell signatures on overall survival of uveal melanoma patients.

Kaplan-Meier plots for (A) NK cell signature 1 (Böttcher et. al), (B) NK cell signature 2 (Cursons et. al) and (C) NK cell signature 3 (Zheng et. al) on overall survival of uveal melanoma patients within the TCGA cohort (n=80). Log-rank test was used to test for significance and hazard ratio (HR) calculations.


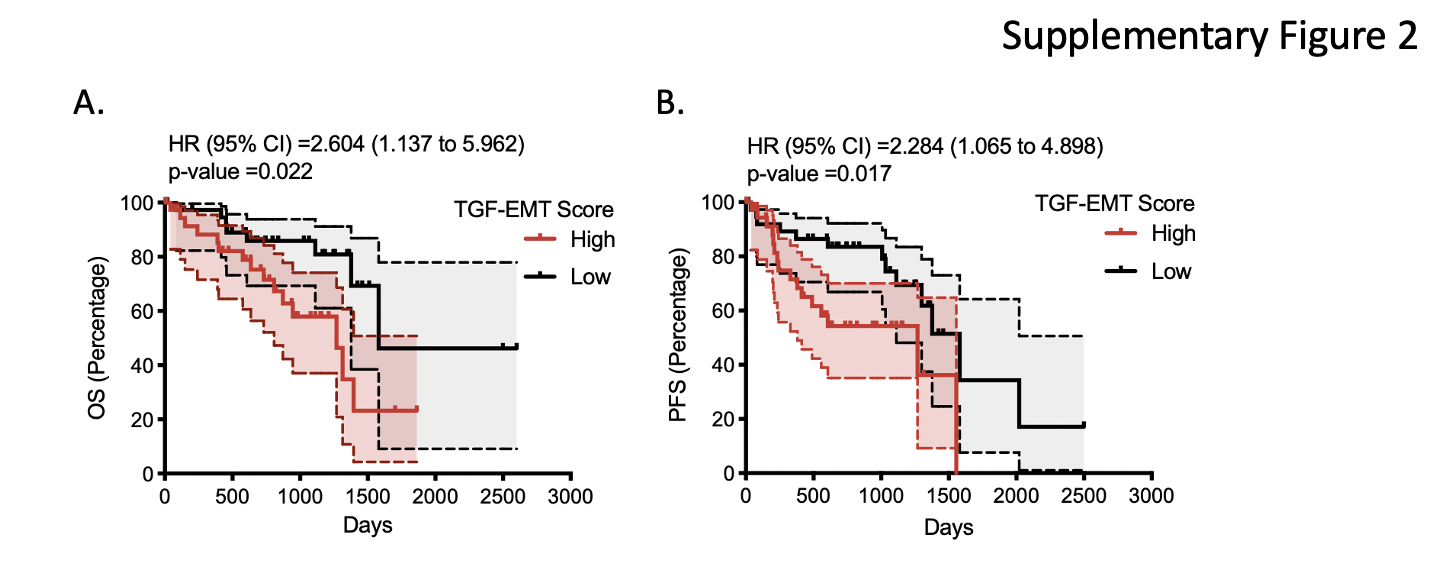
Supplementary figure 2. Prognostic values of a TGF-EMT signature on survival of uveal melanoma patients.

Kaplan Meier plots for (A) Overall survival and (B) Progression-free survival of uveal melanoma patients grouped based on the median of TGF-EMT score within the TCGA cohort (n=80). Log-rank test was used to test for significance and hazard ratio (HR) calculations.


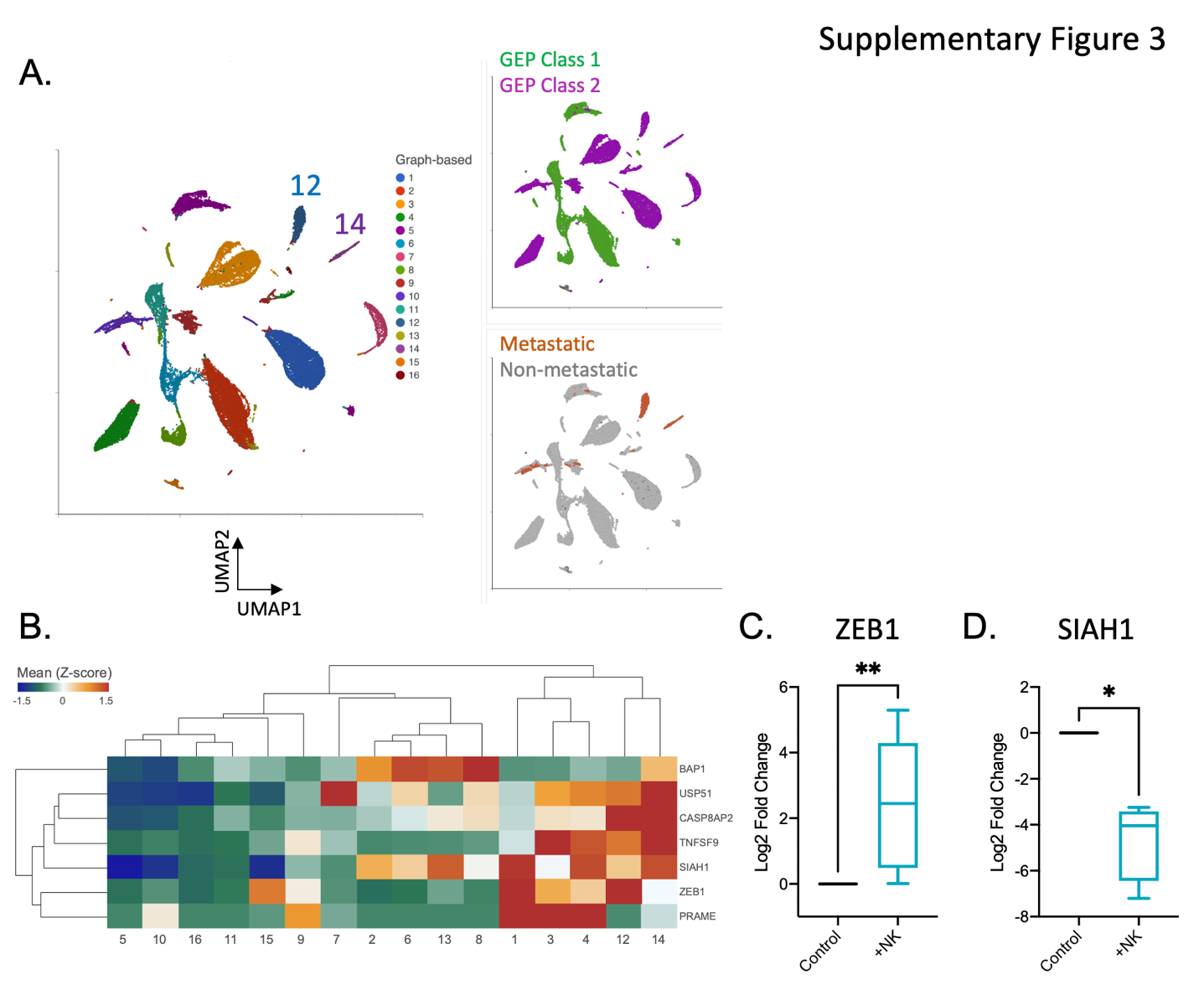
Supplementary Figure 3. Gene expression profiling of uveal melanoma cells

(A) UMAP projection of Louvain clusters (Left), tumor gene expression profiling (GEP) class (Top Right) and metastasis status (Bottom Right). Tumor cells from metastatic cases were identified as clusters 12 and 14. (B) Unsupervised heatmap clustering of BAP1, PRAME. TNFSF9, ZEB1 with known post-translational regulators for ZEB1 ubiquitination. (C) Normalized gene expression of SIAH1 in OCM3 cells after 3 days co-culture with NK cells. Normalized gene expression of (C) ZEB1 and (D) SIAH1 in OCM3 tumor cells after 3 days co-culture with NK cells (n=5).

Supplemental Table 1. Components for liver organoid culture media

| Reagent | Brand | Catalogue number | Concentration | |
| --- | --- | --- | --- | --- |
|  |  |  | Stock | Working |
| Advanced DMEM/F12 | Gibco | 12634010 | 1 X | 1 X |
| GlutaMAX | Gibco | 35050061 | 100 X | 1 X |
| HEPES | Gibco | 15630080 | 1 M | 10 mM |
| Penicillin-Streptomycin | Gibco | 15070063 | 100 X | 1 X |
| N2 | Gibco | 17502048 | 100 X | 1 X |
| B27 | Gibco | 12587010 | 50 X | 1 X |
| N-acetyl-l-cysteine | Sigma | A8199 | 500 mM | 1.25 mM |
| Gastrin | Sigma | G9145 | 100 μM | 10 nM |
| Forskolin | Tocris | 1099 | 25 mM | 10 μM |
| A83-01 | Sigma | SML0788 | 5 mM | 5 μM |
| ^Nicotinamide | Sigma | N0636 | 1 M | 10 mM |
| EGF | PeproTech | 100-15 | 50 μg/ml | 50 ng/ml |
| R-Spondin-1 | R&D SYSTEMS | 4645-RS | 100 μg/ml | 100 ng/ml |
| FGF10 | PeproTech | 100-26 | 100 μg/ml | 100 ng/ml |
| HGF | PeproTech | 100-39H | 25 μg/ml | 25 ng/ml |
| *Y27632 | StemCell Technologies | 72304 | 10 mM | 10 μM |
| *Noggin | PeproTech | 120-10C | 25 μg/ml | 25 ng/ml |
| *Wnt3a | PeproTech | 315-20 | 100 μg/ml | 100 ng/ml |

* Reagents included in the medium during the first three days of culture only.

Supplemental Table 2. Antibodies for Immunofluorescence

| Antigen | Host | Supplier | Catalogue number | Dilution |
| --- | --- | --- | --- | --- |
| SOX9 | Mouse | Abcepta | AT4001a | 1:100 |
| LGR5 | Rabbit | Abgent | AP2745D-EV | 1:100 |
| HNF4a | Rabbit | BioSite | ASJ-BF9TOM | 1:50 |
| KRT19 | Rabbit | BioSite | ASJ-H77D6S | 1:100 |
| E-Cadherin | Mouse | Abcam | ab231303 | 1:200 |
| Anti-rabbit Alexa 555 | donkey | ThermoFisher Scientific | A-31572 | 1:200 |
| Anti-mouse Alexa 647 | donkey | ThermoFisher Scientific | A-31571 | 1:200 |
| YO-PRO™-1 Iodide (491/509) | N.A. | ThermoFisher Scientific | Y3603 | 1:200 |

Supplemental table 3. Antibodies for Flow Cytometry

| Antigen | Fluorochrome | Clone | Company | Catalog no. |
| --- | --- | --- | --- | --- |
| ZEB-1 | Alexa Fluor-647 | E2G6Y | CST | 40098 |
| KI67 | BV785 | B56 | BD Biosciences | 563756 |
| NKP46 | V450 | 9E2/NKp46 | BD Biosciences | 562099 |
| CD45 | BV650 | HI30 | Biolegend | 304044 |
| PD-L1 | BV785 | 29E.2A3 | Biolegend | 329736 |
| IL-10 | APC | Cytokine Kit | Miltenyi | 130-090-434 |
| NKG2D | PE-CF594 | 1D11 | BD Biosciences | 562498 |
| CD73 | APC-Fire 750 | AD2 | Biolegend | 344036 |
| LAG3 | FITC | 11C3C65 | Biolegend | 369326 |
